# Coronary microvascular function, insulin sensitivity and body composition in predicting exercise capacity in overweight patients with coronary artery disease

**DOI:** 10.1186/s12872-015-0151-9

**Published:** 2015-11-27

**Authors:** Anders Jürs, Lene Rørholm Pedersen, Rasmus Huan Olsen, Martin Snoer, Elizaveta Chabanova, Steen Bendix Haugaard, Eva Prescott

**Affiliations:** Department of Cardiology, Bispebjerg Hospital, University of Copenhagen, Copenhagen, Denmark; Department of Radiology, Herlev Hospital, University of Copenhagen, Copenhagen, Denmark; Department of Internal Medicine, Amager Hospital and the Clinical Research Center, Hvidovre Hospital, University of Copenhagen, Copenhagen, Denmark

**Keywords:** Coronary flow reserve, Coronary artery disease, Exercise capacity, Insulin sensitivity, Body composition

## Abstract

**Background:**

Coronary artery disease (CAD) has a negative impact on exercise capacity. The aim of this study was to determine how coronary microvascular function, glucose metabolism and body composition contribute to exercise capacity in overweight patients with CAD and without diabetes.

**Methods:**

Sixty-five non-diabetic, overweight patients with stable CAD, BMI 28–40 kg/m^2^ and left ventricular ejection fraction (LVEF) above 35 % were recruited. A 3-hour oral glucose tolerance test was used to evaluate glucose metabolism. Peak aerobic exercise capacity (VO_2_peak) was assessed by a cardiopulmonary exercise test. Body composition was determined by whole body dual-energy X-ray absorptiometry scan and magnetic resonance imaging. Coronary flow reserve (CFR) assessed by transthoracic Doppler echocardiography was used as a measure of microvascular function.

**Results:**

Median BMI was 31.3 and 72 % had impaired glucose tolerance or impaired fasting glucose. VO_2_peak adjusted for fat free mass was correlated with CFR (r = 0.41, *p* = 0.0007), LVEF (r = 0.33, *p* = 0.008) and left ventricular end-diastolic volume (EDV) (r = 0.32, *p* = 0.01) while it was only weakly linked to measures of glucose metabolism and body composition. CFR, EDV and LVEF remained independent predictors of VO_2_peak in multivariable regression analysis.

**Conclusion:**

The study established CFR, EDV and LVEF as independent predictors of VO_2_peak in overweight CAD patients with no or only mild functional symptoms and a LVEF > 35 %. Glucose metabolism and body composition had minor impact on VO_2_peak. The findings suggest that central hemodynamic factors are important in limiting exercise capacity in overweight non-diabetic CAD patients.

## Background

Randomized and observational studies have shown that exercise capacity is a strong predictor of cardiovascular prognosis. Even when fully revascularized, patients with stable coronary artery disease (CAD) and normal left ventricular ejection fraction (LVEF) have lower than expected exercise capacity [1]. Patients with visceral obesity or the metabolic syndrome also have impaired exercise capacity [2]. The limiting factors of exercise capacity in these clinical entities is unclear.

Reduced coronary microvascular function has emerged as a strong predictor of poor prognosis. In patients with CAD, in patients with diabetes [3] and in subjects with cardiometabolic risk factors, such as hypertension [4] and visceral obesity [5], coronary microvascular function is impaired. Coronary microvascular dysfunction has also been linked to impaired exercise capacity. We have previously found that coronary flow reserve (CFR) as a measure of coronary microvascular function is strongly associated with exercise capacity in CAD and chronic heart failure [6]. We have also described an association between insulin sensitivity and CFR in heart failure patients [7]. An understanding of how these important markers interact in explaining limitations to exercise capacity is lacking. This study explores whether measures of glucose metabolism and body composition contribute to the previously observed link between coronary microvascular function and exercise capacity in overweight non-diabetic patients with CAD.

## Methods

### Study population

This study is based on baseline data from the randomized, controlled CUT-IT trial comparing the cardiovascular effects of a weight loss facilitated using a low energy diet (LED) and aerobic interval training in overweight non-diabetic patients with CAD. The study is in compliance with the Declaration of Helsinki and has been approved by the Regional Ethics Committee of the Capital Region in Denmark (no H-4-2010-146) and the Danish Data Protection Agency (no 2011-41-6313). The trial is registered at clinicaltrials.gov (NCT01724567). Informed written consent for participation in the study was obtained from all participants. The inclusion criteria and study design have been published previously [8, 9]. In summary, inclusion criteria were CAD diagnosed more than 6 months prior to inclusion, age 45 – 75 years, body mass index (BMI) 28–40 kg/m2 and no diabetes. A subgroup of the patients (*n* = 30) were included in a previously reported analyses of association between CFR and VO_2_peak [6]. The present analyses expand those results to a larger patient group and aims at gaining mechanistic insight through including detailed data on body composition and glucose metabolism.

### Cardiopulmonary exercise test

The participants underwent a cardiopulmonary exercise test (CPET) using an upright bicycle ergometer (Via Sprint 150P, Ergoline, Bitz, Germany) with breath-by-breath gas exchange measurement of oxygen consumption and CO_2_ production (Jaeger, Masterscreen CPX, Cardinal Health, Würzburg, Germany) at the screening and the baseline visit. The screening visit was used to familiarize the participant to the test and to ensure that the optimal exercise protocol was used at baseline. The data used in the present study is the baseline test. All participants were encouraged to continue until exhaustion and a satisfactory effort was anticipated when VO_2_ and/or heart rate failed to increase with further increases in workload or peak respiratory exchange ratio (RER) (VCO_2_/VO_2_) exceeded 1.05. VO_2_peak and RER were calculated as the means of the six highest consecutive five-second measurements before exercise termination. VO_2_peak was adjusted to both total body weight (VO_2_peak_BW_) (mL/kg body weight/min) and fat free mass (FFM) (VO_2_peak_FFM_) (mL/kg FFM^(2/3)^/min) [10]. Predicted VO_2_peak was calculated using the equation for sedentary individuals as proposed by Wassermann and Hansen [11].

### Body composition

The participants’ fasting-state body mass, hip and waist circumference were measured by standardized procedures [9]. Body composition, including fat mass and fat free mass, was assessed using whole body dual-energy X-ray absorptiometry (DEXA) (Lunar DPX-IQ, GE Lunar Corp, Madison, WI). The ratio of central to peripheral distribution of body fat was estimated by dividing the body fat in the DXA trunk region of interest (ROI) by the ROIs that encompass the arms, hips and legs [12]. Visceral abdominal adipose tissue was determined by magnetic resonance imaging (MRI) in a subgroup (*n* = 37). MRI measurements and analyses were performed using the Achieva 3.0 T MRI system and the Philips ViewForum workstation (Philips Medical Systems, Best, the Netherlands) and a sense cardiac coil. Images were obtained by a fast T1w turbo field echo (TFE) MR sequence in the transverse plane (TFE sequence, TFE factor = 136, TR = 10 ms, TE = 2.3 ms, FOV = 480 mm, respiratory trigger compensation with trigger delay of 1000 ms). A transverse slice (thickness: 10 mm) was acquired in the supine position in the middle of the third lumbar vertebra and visceral fat was measured.

### Oral glucose tolerance test

A 3 hour oral glucose tolerance test (OGTT) with measurement of plasma glucose-, insulin- , and C-peptide levels was performed. After a 10 hour overnight fast an oral glucose load of 75 g dissolved in 300 ml water was administered within 2 minutes. Plasma samples were obtained at −10,0,10,20,30,45,60,75,90,105,120,150 and 180 minutes. Blood samples were immediately cooled on ice and centrifuged for 10 minutes (3500 rpm, Universal 320R, Hettich Centrifugen, Tuttlingen, Germany). Glucose analysis was performed using an YSI 2300 STAT Plus Glucose and Lactate Analyser (YSI incorporated, Yellow Springs, OH, USA). Enzyme-linked immunosorbent assay (ELISA) was used to determine C-peptide and insulin (pmol/l, Immulite 2000, Siemens Healthcare Diagnostics, LA, Califonia, USA). Glycated hemoglobin HbA1c was estimated by high performance liquid chromatography.

Based on the OGTT the following glucose metabolic parameters were calculated:

Fasting plasma glucose (FPG, mmol/L) and fasting plasma insulin (FPI, pmol/L) were calculated as the average of plasma values at time −10 and 0 minutes. Glucose tolerance was defined as the 2 hour plasma glucose concentration (2 h-PG). The composite measure of whole body insulin sensitivity (ISI_Composite_) [13] and the homeostatis model assessment of hepatic insulin resistance (HOMA-IR) [14] were determined as follows:

$$ IS{I}_{composite}=\frac{10.000}{\sqrt{\left(FPG\times FPI\right)\times \left(\overline{G}\times \overline{I}\right)}}\kern1.2em  HOMA-IR=\frac{\left(FPG\times FPI\right)}{405} $$

in which FPG is fasting plasma glucose (mg/dl), FPI is fasting plasma insulin (μU/ml), $\overline{G}$ and $\overline{I}$ is mean plasma glucose and insulin, respectively, measured during the OGTT at 0, 30, 60, 90 and 120 minutes. 1/HOMA-IR was used as an estimation of insulin sensitivity (ISI_HOMA_).

Prehepatic insulin secretion rates (ISR) (pmol/kg/min) were calculated from plasma C-peptide concentrations using the ISEC (Insulin SECretion) computer program [15]. The method is based upon the assumptions that insulin and C-peptide are co-secreted in equimolar amounts by the pancreas and that C-peptide is not cleared by the liver. β-Cell secretion in response to changes in glucose concentration during the OGTT expresses the efficacy by which changes in plasma glucose concentrations stimulate insulin secretion. The relationship between plasma glucose concentrations and ISR during the OGTT was evaluated by cross-correlation analysis and the slope of the regression lines (β_total_), which express the change in insulin secretion per unit change in glucose concentration, was used as a measure of β-cell responsiveness. Disposition index (Di) was calculated as a measure of β-cell function adjusted for insulin sensitivity [16]:

$$ D{}_i={\beta}_{total}\times {\mathrm{ISI}}_{composite} $$

### Coronary flow reserve and echocardiography

All participants underwent a complete transthoracic echocardiography using a Vivid E9 (GE Medical Systems, Inc., Horten, Norway). Left ventricular end-diastolic volume (EDV), end-systolic volume (ESV) and left ventricular ejection fraction (LVEF) were calculated using the biplane Simpson method. EDV and ESV were corrected for body surface area using the Mosteller formula [17]. CFR was measured using a high frequency S6 transducer (GE Medical Systems, Inc., Horten, Norway). All participants were instructed to abstain from caffeine 24 hours prior to the examination and the use long acting nitroglycerin was suspended for 24 hours. Starting in a modified apical 5-chamber view, which intersected the anterior wall, and using color Doppler with a low Nyquist limit, the left anterior descending artery (LAD) was located in its distal path. If unsuccessful the LAD was located mid-distally in the interventricular sulcus using a low short-axis view. Hyperemia was induced by infusion of dipyridamole (0.84 mg/kg over 6-minutes) or adenosine (0.14 mg/kg/min for 2 minutes) . CFR was calculated as the ratio between peak coronary flow velocity at rest and during hyperemia. We have previously reported inter and intra-observer variability of repeated off-line CFR readings with within-subject coefficient of variation (CV) of 5.5 % (*n* = 39) and 7.5 % (*n* = 10), respectively [18].

### Statistics

Unless stated otherwise all values are expressed as median and interquartile range for continuous variables and as number and percentage for categorical variables. Continuous variables were compared using Student’s t-test and differences in categorical variables were assessed by χ^2^ test. The Pearson product–moment correlation coefficient was used to estimate the associations between continuous variables and multivariable linear regression with standardized coefficients (SC) were performed to identify independent predictors of VO_2_peak. The multivariable model included age and gender (fixed) and tested each covariate based on associations from univariate analyses as specified below. Variables with p < 0.10 were retained in the multivariable model. The significance level was set to p < 0.05. All analyses were done using Stata 13.1 software (StataCorp, College Station, TX, USA).

## Results

We recruited 70 participants without significant stenosis of the LAD. Of these 65 participants completed a satisfactory CPET and had a successful CFR measurement. In two patients the image quality was too poor to assess CFR and three patients did not fulfill the criteria for a satisfactory CPET. Patient characteristics are presented in Table 1. Patients were primarily male and had well-regulated blood pressures. Fifty-seven (87.7 %) participants had previously been revascularized, and none had ischemic changes in ECG during CPET. Thirteen (20 %) participants reported angina only during strenuous or prolonged physical activity while the remaining participants were asymptomatic. Median BMI was 31.3 (29.7-33.7) and forty-seven (72 %) of the patients had prediabetes defined as either impaired glucose tolerance (FPG < 7.0 mmol/L and 7.8 ≤ 2hPG < 11.1 mmol/L) or impaired fasting glucose (FPG 5.6-6.9 mmol/L).

**Table 1:** Patient characteristics. Values are median (IQ range) or number (%)

Patient characteristics *n* = 65	Median/number	IQ range/percentage
Age	63	(58–67)
Male Sex	54	(83.1 %)
Fat percentage (%)	34.0	(30.5–38.6)
BMI (kg/m^2^)	31.3	(29.7–33.7)
Prediabetes	47	(72.3 %)
Predicted VO_2_peak (%)	93.9 %	(84.6–109.2)
VO_2_peak_BW_ (ml/min/kg)	20.9	(17.3–25.1)
VO_2_peak_FFM_ (ml/min/kg FFM^(2/3)^)	127	(108.8–145.6)
Respiratory exchange ratio	1.19	(1.16–1.26)
ICD	2	(3.1 %)
Systolic blood pressure (mmHg)	127	(118–135)
Diastolic blood pressure (mmHg)	72	(67–79)
Medication		
* -Beta-blockers* * -Statins* * -ASA or/and Clopidogrel* * -Diuretics* * -ACE-inhibitors*	34 63 62 21 29	(52.3 %) (96.9 %) (95.4 %) (32.3 %) (44.6 %)
Atrial fibrillation	3	(4.6 %)
Left ventricular ejection fraction (%)	54	(46–59)
Ischemic etiology		
* -Previous PCI or CABG* * Involving LAD* * -Previous myocardial infarction* * Involving LAD*	57 36 35 15	(87.7 %) (55.4 %) (53.9 %) (23.1 %)
CCS-class		
-|	13	(20.0 %)
NYHA-class		
-| -|| -|||	50 14 1	(76.9 %) (21.5 %) (1.5 %)

*BMI* Body mass index, *ICD* Implantable cardioverter-defibrillator, *ASA* acetylsalicylic acid, *ACE* Angiotensin converting enzyme, *PCI* percutaneous coronary intervention, *CABG* coronary artery bypass graft, *LAD* left anterior descending artery, *CCS* Canadian Cardiovascular Society Functional Classification of Angina Pectoris, *NYHA* New York Heart Association, *VO*_*2*_ *peak*_*BW*_ peak rate of oxygen consumption per kilogram of body mass, *VO*_*2*_ *peak*_*FFM*_ peak rate of oxygen consumption per kilogram of fat free body mass

No differences were found in patient characteristics between patients with or without a successful CPET or CFR measurement.

### Coronary flow reserve and echocardiography

Median CFR was 2.28 (1.91-2.61) and median LVEF was 54 % (47–59). There was no significant difference in CFR in participants with and without an ischemic etiology involving the LAD, by NYHA-class or gender. Table 2 shows measures of echocardiography, exercise capacity, glucose metabolism and body composition by CFR above and below the median CFR. Patients with a high CFR had a higher VO_2_peak_FFM_ (*P* = 0.0008) and a lower body fat percentage (*P* = 0.02). Results from univariate regression on CFR are presented in Table 3 and confirms a strong association between CFR and VO_2_peak_FFM_ (*p* = 0.0007).

**Table 2:** Patient´s measurements divided into “low” or “high” groups according to median CFR and VO_2_peak_FFM_

	Coronary flow reserve				VO_2_Peak_FFM_			
	Low (CFR ≤ 2.28)		High (CFR > 2.28)		Low (VO_2_Peak_FFM_ ≤ 126.5)		High (VO_2_Peak_FFM_ >126.5)	
	*n* = 32		*n* = 33		*n* = 32		*n* = 33	
Patient characteristics								
Age	64	(59–68)	62	(56–67)	65	(61–69)	62	(55–65)*
Male sex	26	(81.3 %)	28	(84.9 %)	24	(75.0 %)	30	(90.9 %)
CABG/PCI involving LAD	16	(50.0 %)	20	(60.0 %)	19	(59.4 %)	17	(51.5 %)
ACE–inhibitors	16	(50.0 %)	13	(39.4 %)	16	(50.0 %)	13	(39.4 %)
Beta–blockers	17	(53.1 %)	17	(51.5 %)	17	(53.1 %)	17	(51.5 %)
Echocardiography								
Left ventricular ejection fraction (%)	53	(45–59)	56	(48–60)	54	(46–58)	55	(47–61)
End diastolic volume (mL/m^2^)	38.4	(34.4–50.5)	40.8	(34.5–47.7)	36.0	(31.3–39.6)	43.6	(39.2–54.1)*
End systolic volume (mL/m^2^)	17.6	(15.0–26.6)	17.7	(14.2–24.3)	16.4	(13.8–22.0)	18.5	(15.6–27.0)*
Coronary flow reserve (ratio)	1.91	(1.77–2.09)	2.62	(2.53–2.97)*	2.13	(1.87–2.55)	2.52	(2.07–2.71)*
Exercise capacity								
VO_2_peak_BW_ (ml/min/kg)	18.3	(16.5–21.9)	23.2	(20.5–27.3)*	17.7	(16.0–20.5)	25.1	(22.3–27.6)*
VO_2_peak_FFM_ (ml/min/kg FFM^(2/3)^)	116.0	(104.0–129.9)	135.5	(121.6–161.9)*	108.7	(98.4–119.6)	145.6	(133.1–162.7)*
Respiratory exchange ratio (RER)	1.21	(1.16–1.28)	1.19	(1.15–1.22)	1.21	(1.15–1.28)	1.18	(1.15–1.23)
Glucose metabolism								
Insulin sensitivity (ISI_composite_)	2.21	(1.71–3.50)	2.49	(1.76–3.59)	2.28	(1.88–3.35)	2.48	(1.66–3.77)
Insulin sensitivity (ISI_HOMA_)	0.28	(0.19–0.46)	0.31	(0.22–0.46)	0.28	(0.19–0.45)	0.30	(0.22–0.48)
Hba1c (%)	5.9	(5.7–6.1)	5.9	(5.5–6.2)	6.1	(5.9–6.3)	5.8	(5.5–6.0)*
Fasting plasma glucose (mmol/L)	5.88	(5.31–6.33)	5.85	(5.65–6.18)	6.07	(5.70–6.33)	5.73	(5.46–6.07)*
Fasting plasma insulin (pmol/L)	100	(60–131)	88	(56–120)	94	(60–124)	93	(61–121)
Glucose tolerance (2 h-PG) (mmol/L)	7.95	(6.31–9.49)	7.37	(5.91–8.86)	8.14	(6.47–9.66)	7.17	(5.64–8.85)
Beta cell function (B_total_) (nL/kg/min)	1.90	(1.46–2.37)	1.93	(1.46–2.86)	1.85	(1.45–2.37)	1.99	(1.46–2.71)
β-cell function adjusted for insulin sensitivity (D_i_)	4.89	(2.82–8.12)	5.52	(3.18–7.45)	5.48	(3.06–8.98)	4.64	(2.82–7.17)
Body composition								
BMI (kg/m^2^)	31.1	(29.5–35.1)	31.5	(29.9–33.3)	31.0	(29.5–33.2)	31.8	(29.9–33.7)
Waist-to-hip-ratio	1.00	(0.98–1.03)	1.01	(0.97–1.04)	1.00	(0.96–1.05)	1.00	(0.97–1.03)
Body fat (%)	35.7	(32.6–41.0)	32.2	(29.1–35.7)*	34.3	(31.0–42.1)	33.4	(30.5–35.8)
Visceral fat (mm^3^)	293	(260–332)(*n* = 14)	276	(211–316)(*n* = 23)	293	(222–345)(*n* = 16)	280	(211–297)(*n* = 21)
Central-to-peripheral fat ratio	1.52	(1.34–1.70)	1.65	(1.39–2.05)	1.54	(1.34–1.82)	1.59	(1.39–1.89)

*VO*_*2*_ *peak*_*FFM*_ peak rate of oxygen consumption per kilogram of fat free body mass, *CABG* coronary artery bypass graft, *PCI* percutaneous coronary intervention, *LAD* left anterior descending artery, *D*_*i*_ disposition index, *BMI* Body mass index, *VO*_*2*_ *peak*_*BW*_ peak rate of oxygen consumption per kilogram of body mass. *p < 0.05

**Table 3:** Univariate analysis of correlations to CFR and VO_2_peak_FFM_

	Univariate correlations			
	Coronary flow reserve		VO_2_Peak_FFM_	
	r	*p*-value	r	*p*-value
Age	−0.13	0.30	−0.38	0.0015
Echocardiography				
Left ventricular ejection fraction (%)	0.20	0.10	0.33	0.008
End diastolic volume (mL/m^2^)	−0.06	0.61	0.32	0.01
End systolic volume (mL/m^2^)	−0.10	0.44	0.13	0.30
Coronary flow reserve (ratio)	-	-	0.41	0.0007
Exercise capacity				
VO_2_peak_FFM_ (ml/min/kg FFM^(2/3)^)	0.41	0.0007	-	-
Respiratory exchange ratio (ratio)	−0.17	0.17	−0.08	0.52
Glucose metabolism				
Insulin sensitivity (ISI_composite_)	0.04	0.73	0.08	0.53
Insulin sensitivity (ISI_HOMA_)	0.06	0.65	0.16	0.18
Hba1c (%)	0.03	0.79	−0.30	0.014
Fasting plasma glucose (mmol/L)	−0.06	0.64	−0.22	0.08
Fasting plasma insulin (pmol/L)	−0.16	0.20	−0.10	0.46
Glucose tolerance (2 h-PG) (mmol/L)	−0.20	0.09	−0.20	0.10
Beta cell function - B_total_ (nL/kg/min)	0.11	0.36	−0.03	0.84
β-cell function adjusted for insulin sensitivity (D_i_)	0.23	0.07	0.08	0.56
Body composition				
BMI (kg/m^2^)	−0.15	0.22	−0.05	0.67
Waist-to-hip-ratio	0.07	0.59	0.02	0.88
Body fat (%)	−0.21	0.09	−0.29	0.02
Visceral fat (mm^3^) (*n* = 37)	−0.22	0.17	−0.28	0.10
Central-to-peripheral fat ratio	0.13	0.30	−0.18	0.16

*VO*_2_ *peak*_*FFM*_ peak rate of oxygen consumption per kilogram of fat free body mass, *D*_*i*_ disposition index, *BMI* Body mass index

Insulin sensitivity, as defined by the parameters ISI_HOMA_ and ISI_Composite_ showed no association with CFR. Neither did β-cell function (β_total_), nor glucose tolerance as defined by 2 h-PG. However, when grouping participants by impaired coronary microvascular function (defined as CFR < 2) or not, glucose tolerance was significantly worse in participants with impaired coronary microvascular function (*p* = 0.008).

### Exercise capacity

Though not significant, the patients had lower than predicted exercise capacity with a median VO_2_peak_bw_ of 21.3 mL/min/kg (17.5-25.1) and a median VO_2_peak % of predicted of 93.9 % (*p* = 0.12). RER values indicated that participants performed a satisfactory CPET. Table 2 compares echocardiographic measures, measures of glucose metabolism and body composition across patients with low and high exercise capacity dichotomized by median VO_2_peak_FFM_. Patients with a higher exercise capacity were younger, had larger left ventricular volumes and better CFR (Table 2).

Measures of glucose metabolism were numerically better in the patients with higher VO_2_peak_FFM_ but only for FPG and Hba1c did the difference reach statistical significance (Tables 2 and 3). As expected, HOMA-IR, ISI_Composite_ and D_i_ were, along with the glucose and insulin measurements on which they were calculated, significantly correlated with Hba1c, MCRi and visceral fat while the correlation to body fat percentage was borderline-significant (data not shown). In general participants with high VO_2_peak_FFM_ had a lower body fat percentage, less visceral fat and less abdominal fat (Tables 2 and 3). Only body fat percentage reached statistical significance; however, only 37 patients underwent MRI.

Results of univariate linear regression analyses in relation to VO_2_peak_FFM_ are presented in Table 3. VO_2_peak_FFM_ was significantly correlated with LVEF, CFR, EDV, and Hba1c. VO_2_peak_BW_ showed similar correlations.

Independent predictors of VO_2_peak were identified using a multivariable regression model presented in Table 4. Age and gender, which are traditional predictors VO_2_peak, were included along with the significant correlates from the univariate analysis presented in Table 3. CFR, LVEF and EDV remained independent predictors of exercise capacity while Hba1c was no longer significant.

**Table 4:** Multivariable linear regression with VO2peak as the dependent variable

	VO_2_peak_FFM_			VO_2_peak_BW_	
	SC	*p*-value		SC	*p*-value
Age	−0.14	0.204	Age	−0.08	0.370
Male sex	0.17	0.226	Male sex	0.01	0.901
Body fat %	−0.01	0.951	Body fat %	−0.40	0.002
Coronary flow reserve (ratio)	0.32	0.003	Coronary flow reserve (ratio)	0.29	0.002
Left ventricular ejection fraction (%)	0.37	0.001	Left ventricular ejection fraction (%)	0.32	0.001
Left ventricular end diastolic volume (mL/m^2^)	0.33	0.005	Left ventricular end diastolic volume (mL/m^2^)	0.29	0.005
Hb1ac (%)	−0.18	0.075	Hb1ac (%)	−0.17	0.063

*SC* Standardized coefficient, *VO*_*2*_ *peak*_*FFM*_ peak rate of oxygen consumption per kilogram of fat free body mass, *VO*_*2*_ *peak*_*BW*_ peak rate of oxygen consumption per kilogram of body mass. Respectively 48 % and 59 % of the variance of VO_2_peak_FFM_ and VO_2_peak_BW_ was explained by the included variables

ACE-inhibitors and beta-blockers can influence exercise capacity and CFR. The distribution of these medications between participants with high and low CFR and high and low VO_2_peak_FFM_ is shown in Table 2. There were no significant differences. Adjustment for the use of beta-blockers and/or ACE-inhibitors did not affect the association between CFR and VO_2_peak_FFM_.

**Fig. 1:**
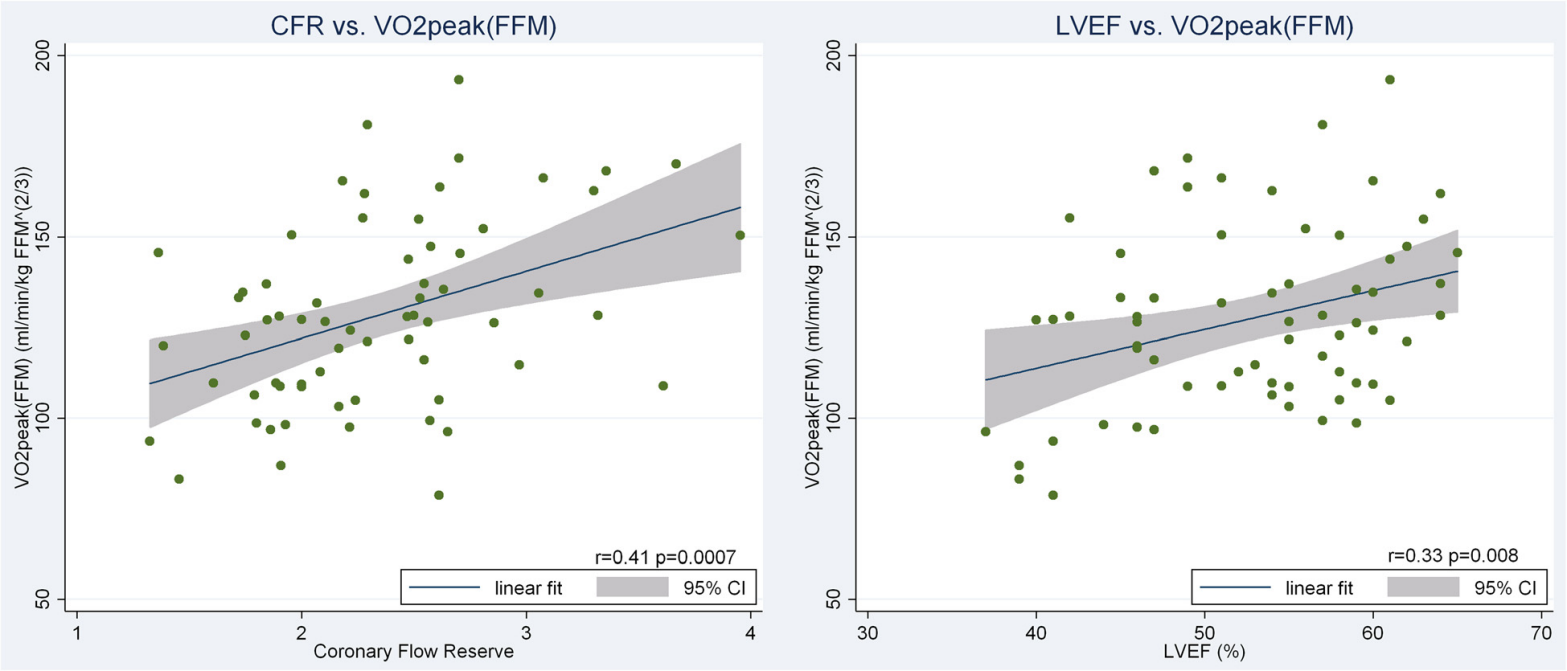
Scatter plots with linear regression lines and confidence interval (CI) between VO_2_peak_FFM_ and CFR (left) and VO_2_peak_FFM_ and LVEF (right)

CFR measurement is performed in the LAD. Adjustment for involvement of LAD (PCI, CABG or MI) and limiting analyses to patients with no involvement of the LAD did not affect estimates. Figure 1 shows scatterplots with linear regression lines between VO_2_peak_FFM_ and CFR and VO_2_peak_FFM_ and LVEF. Results were similar when using VO_2_peak_BW_ as the dependent variable.

## Discussion

The present study is the first to investigate the effect of glucose metabolism and body composition on coronary microvascular function and exercise capacity in non-diabetic overweight patients with CAD. Our results confirm a strong association between coronary microvascular function and exercise capacity, but indicate that the role of glucose metabolism and body composition is limited in non-diabetic, overweight CAD patients and that the reduced exercise capacity is primarily determined by other parameters.

### Microvascular function

Two studies found a correlation between insulin sensitivity measured by the hyperinsulinemic-euglycemic clamp technique and CFR in heart failure patients (*n* = 39) [7] and in obese subjects (*n* = 36 ) [19], respectively. A study including non-diabetic women with suspected CAD but angiographically normal coronary arteries (*n* = 45) found a correlation between CFR and insulin resistance using an OGTT [20]. In the present study insulin sensitivity and appropriate β-cell function as measured by disposition index were congruent with higher CFR, but associations were non-significant. This may be due to the methods used. In the present study insulin sensitivity was evaluated by an OGTT with 13 plasma samples obtained over a 3 hour period. The clamp technique is considered to be the gold standard, however, all indices from the OGTT have been validated against the clamp technique and are regarded as valid measures [13, 14]. Also, the oral test may be considered more physiologic than the intravenous clamp technique by activating the important incretin effects. However, when considering the glucose tolerance in those 40 % of patients who by definition display impaired CFR (<2.0) they displayed a highly significant impaired glucose tolerance compared to those 60 % of patients with normal CFR. Thus data available from previous studies and the present study appear slightly inconsistent, but notably the number of studies is limited.

CFR is a measure of coronary microvascular function and body composition has previously been linked to peripheral endothelial function [21] and the endothelium-related myocardial blood flow response as measured by PET (*n* = 111) [22]. Therefore an association between CFR and body composition could be expected. Participants with low CFR had a significantly higher body fat percentage; however, the measures of body composition were not associated with CFR in the regression analyses. Only 37 participants underwent MRI and consequently the study may have been under-powered to assess associations between visceral fat and CFR.

### Exercise capacity

The achieved respiratory exchange ratios achieved during exercise testing indicate that all patients delivered a satisfactory CPET. The equal median RER-values in the groups of high and low exercise capacity further accentuate that the VO_2_peak_FFM_ was not influenced by exercise effort.

HbA1c and FPG demonstrated significant inverse associations to VO_2_peak_FFM_ and estimates of insulin sensitivity, glucose tolerance and β-cell function showed a trend to associate to better exercise capacity. These findings are only partly in concordance with previous studies. Studies evaluating the correlation of exercise capacity and insulin sensitivity using the glucose clamp technique in a healthy population (*n* = 20 ) [23] and heart failure patients (*n* = 39 and *n* = 9 , respectively) [7, 24] reported an association between insulin sensitivity and VO_2_peak. A recent study of 170 patients with CAD and diabetes also found VO_2_peak to be correlated with HOMA-IR but not with FPG or Hba1c [25] while another study evaluating the effect of exercise training in a similar population found a correlation between baseline FPG and Hba1c and VO_2_peak but not between HOMA-IR and VO_2_peak [26].

Thus, our results indicate that the effect of the glucose metabolism on coronary microvascular function is limited and does not explain the previously observed link between CFR and exercise capacity in CAD patients without diabetes. In this group of patients, exercise capacity was primarily determined by other parameters than body composition and glucose metabolism. These parameters included left ventricular ejection fraction and end diastolic volume.

Although cardiac output is defined by stroke volume, which in turn is determined by end diastolic volume and ejection fraction, previous studies have not found this straight-forward association. A large study investigating the relationship in patients with a clinical indication for exercise echocardiography, no exercise-induced myocardial ischemia and LVEF > 50 % found no correlation between LVEF and exercise capacity [27]. However, exercise capacity was calculated from the exercise echocardiography using metabolic equivalents, which is less accurate compared to the CPET used in the present study. Most studies in heart failure patients found little or no association between LVEF and VO_2_peak [18, 28, 29] whereas a study investigating the relationship in asymptomatic CAD patients (*n* = 27) described a correlation between peak LVEF and exercise capacity [30].

Like previous studies in healthy subjects [31, 32], heart failure patients [7, 18] and CAD patients [6], we found a strong correlation between CFR and exercise capacity. Exercise capacity is a strong prognostic marker in patients with CAD [1] and it has been demonstrated that exercise training can improve coronary endothelial function in CAD patients [33]. In this study population consisting of overweight CAD patients with a LVEF > 35 % and primarily NYHA class I or II, CFR remained an independent predictor of VO_2_peak_FFM_ in the multivariable linear regression analysis alongside LVEF and EDV. This is, to our knowledge, the largest study evaluating the correlation of CFR and exercise capacity and the first study establishing CFR and LVEF to be independent predictors of VO_2_peak.

## Conclusion

The study established that coronary microvascular function and LVEF are independent predictors of VO_2_peak in overweight CAD patients with no or only mild functional symptoms and a LVEF > 35 %. Glucose metabolism and body composition were weak determinants of exercise capacity and did not contribute significantly to explain the link between CFR and exercise capacity. The findings suggest that central hemodynamic factors are important in limiting exercise capacity in overweight non-diabetic CAD patients.

## Abbreviations

CAD: coronary artery disease
LVEF: left ventricular ejection fraction
CFR: coronary flow reserve
EDV: end-diastolic volume
LED: low energy diet
CPET: cardiopulmonary exercise test
RER: respiratory exchange ratio
BW: body weight
FFM: fat free mass
DEXA: dual-energy X-ray absorptiometry
MRI: magnetic resonance imaging
OGTT: oral glucose tolerance test
FPG: fasting plasma glucose
FPI: Fasting plasma insulin
2 h-PG: 2-hour plasma glucose
ISI_composite_: composite measure of whole body insulin sensitivity
HOMA-IR: homeostatis model assessment of hepatic insulin resistance
ISR: insulin secretion rates
β_total_: β-cell responsiveness
Di: disposition index
ESV: end-systolic volume
LAD: left anterior descending artery
SC: standardized coefficients
BMI: body mass index
ICD: implantable cardioverter-defibrillator
ASA: acetylsalicylic acid
ACE: angiotensin converting enzyme
PCI: percutaneous coronary intervention
CABG: coronary artery bypass graft
CCS: Canadian Cardiovascular Society Functional Classification of Angina Pectoris
NYHA: New York Heart Association

## References

[1] Keteyian SJ, Brawner CA, Savage PD, Ehrman JK, Schairer J, Divine G (2008). Peak aerobic capacity predicts prognosis in patients with coronary heart disease. Am Heart J.

[2] Wong CY, O’Moore-Sullivan T, Fang ZY, Haluska B, Leano R, Marwick TH (2005). Myocardial and vascular dysfunction and exercise capacity in the metabolic syndrome. Am J Cardiol.

[3] Yokoyama I, Momomura S, Ohtake T, Yonekura K, Nishikawa J, Sasaki Y (1997). Reduced myocardial flow reserve in non-insulin-dependent diabetes mellitus. J Am Coll Cardiol.

[4] Hamouda MS, Kassem HK, Salama M, El Masry M, Shaaban N, Sadek E (2000). Evaluation of coronary flow reserve in hypertensive patients by dipyridamole transesophageal doppler echocardiography. Am J Cardiol.

[5] Ahmari SA, Bunch TJ, Modesto K, Stussy V, Dichak A, Seward JB (2008). Impact of individual and cumulative coronary risk factors on coronary flow reserve assessed by dobutamine stress echocardiography. Am J Cardiol.

[6] Snoer M, Olsen RH, Monk-Hansen T, Pedersen LR, Haugaard SB, Dela F, et al. Coronary Flow Reserve Predicts Cardiopulmonary Fitness in Patients with Coronary Artery Disease Independently of Systolic and Diastolic Function. Echocardiography. 2013. 10.1111/echo.12445 24299009

[7] Snoer M, Monk-Hansen T, Olsen RH, Pedersen LR, Simonsen L, Rasmusen H (2012). Insulin resistance and exercise tolerance in heart failure patients: linkage to coronary flow reserve and peripheral vascular function. Cardiovasc Diabetol.

[8] Pedersen LR, Olsen RH, Frederiksen M, Astrup A, Chabanova E, Hasbak P (2013). Copenhagen study of overweight patients with coronary artery disease undergoing low energy diet or interval training: the randomized CUT-IT trial protocol. BMC Cardiovasc Disord.

[9] Pedersen LR, Olsen RH, Jürs A, Astrup A, Chabanova E, Simonsen L, et al. A randomised trial comparing weight loss with aerobic exercise in overweight individuals with coronary artery disease: The CUT-IT trial. Eur J Prev Cardiol. 2014. 10.1177/2047487314545280 25082954

[10] von Dobeln W (1956). Maximal oxygen intake, body size, and total hemoglobin in normal man. Acta Physiol Scand.

[11] Guazzi M, Adams V, Conraads V, Halle M, Mezzani A, Vanhees L (2012). EACPR/AHA Joint Scientific Statement. Clinical recommendations for cardiopulmonary exercise testing data assessment in specific patient populations. Eur Heart J.

[12] Clark MK, Dillon JS, Sowers M, Nichols S (2005). Weight, fat mass, and central distribution of fat increase when women use depot-medroxyprogesterone acetate for contraception. Int J Obes (Lond).

[13] Matsuda M, DeFronzo RA: Insulin sensitivity indices obtained from oral glucose tolerance testing: comparison with the euglycemic insulin clamp. Diabetes Care 1999, 22(0149–5992 (Print)):1462–1470. 10.2337/diacare.22.9.1462 10480510

[14] Matthews DR, Hosker JP, Rudenski AS, Naylor BA, Treacher DF, Turner RC: Homeostasis model assessment: insulin resistance and beta-cell function from fasting plasma glucose and insulin concentrations in man. Diabetologia 1985, 28(0012-186X (Print)):412–419. 10.1007/BF00280883 3899825

[15] Hovorka R, Soons PA, Young MA (1996). ISEC: a program to calculate insulin secretion. Comput Methods Programs Biomed.

[16] Bergman RN, Ader M, Huecking K, Van Citters G (2002). Accurate Assessment of -Cell Function: The Hyperbolic Correction. Diabetes.

[17] Mosteller RD (1987). Simplified calculation of body-surface area. N Engl J Med.

[18] Snoer M, Monk-Hansen T, Olsen RH, Pedersen LR, Nielsen OW, Rasmusen H, et al. Coronary flow reserve as a link between diastolic and systolic function and exercise capacity in heart failure. EurHeart JCardiovascImaging 2013, 14(2047–2412 (Electronic)):677–683. 10.1093/ehjci/jes269 23169759

[19] Kondo I, Mizushige K, Hirao K, Nozaki S, Tsuji T, Masugata H (2001). Ultrasonographic assessment of coronary flow reserve and abdominal fat in obesity. Ultrasound Med Biol.

[20] Eroglu S, Sade LE, Bozbas H, Haberal A, Ozbicer S, Demir O (2009). Association of serum adiponectin levels and coronary flow reserve in women with normal coronary angiography. Eur J Cardiovasc Prev Rehabil.

[21] Green DJ, Walsh JH, Maiorana A, Best MJ, Taylor RR, O’Driscoll JG (2003). Exercise-induced improvement in endothelial dysfunction is not mediated by changes in CV risk factors: pooled analysis of diverse patient populations. AmJPhysiol Hear CircPhysiol.

[22] Quercioli A, Pataky Z, Montecucco F, Carballo S, Thomas A, Staub C (2012). Coronary vasomotor control in obesity and morbid obesity: contrasting flow responses with endocannabinoids, leptin, and inflammation. JACC Cardiovasc Imaging.

[23] Botker HE, Frobert O, Moller N, Christiansen E, Schmitz O, Bagger JP (1997). Insulin resistance in cardiac syndrome X and variant angina: influence of physical capacity and circulating lipids. Am Heart J.

[24] Adachi H, Ohno T, Oguri M, Oshima S, Taniguchi K (2007). Effect of insulin sensitivity on severity of heart failure. Diabetes Res Clin Pract.

[25] Byrkjeland R, Edvardsen E, Njerve IU, Arnesen H, Seljeflot I, Solheim S (2014). Insulin levels and HOMA index are associated with exercise capacity in patients with type 2 diabetes and coronary artery disease. Diabetol Metab Syndr.

[26] Verges B, Patois-Verges B, Cohen M, Lucas B, Galland-Jos C, Casillas JM (2004). Effects of cardiac rehabilitation on exercise capacity in Type 2 diabetic patients with coronary artery disease. Diabet Med.

[27] Grewal J, McCully RB, Kane GC, Lam C, Pellikka PA (2009). Left ventricular function and exercise capacity. JAMA.

[28] Gardin JM, Leifer ES, Fleg JL, Whellan D, Kokkinos P, Leblanc M-H (2009). Relationship of Doppler-Echocardiographic left ventricular diastolic function to exercise performance in systolic heart failure: the HF-ACTION study. Am Heart J.

[29] Smart N, Haluska B, Leano R, Case C, Mottram PM, Marwick TH (2005). Determinants of functional capacity in patients with chronic heart failure: role of filling pressure and systolic and diastolic function. Am Heart J.

[30] Ehsani AA, Biello D, Seals DR, Austin MB, Schultz J (1984). The effect of left ventricular systolic function on maximal aerobic exercise capacity in asymptomatic patients with coronary artery disease. Circulation.

[31] Kiviniemi TO, Snapir A, Saraste M, Toikka JO, Raitakari OT, Ahotupa M (2006). Determinants of coronary flow velocity reserve in healthy young men. AmJPhysiol Hear CircPhysiol.

[32] Hagg U, Wandt B, Bergstrom G, Volkmann R, Gan LM (2005). Physical exercise capacity is associated with coronary and peripheral vascular function in healthy young adults. AmJPhysiol Hear CircPhysiol.

[33] Hambrecht R, Wolf A, Gielen S, Linke A, Hofer J, Erbs S (2000). Effect of exercise on coronary endothelial function in patients with coronary artery disease. N Engl J Med.

